# CNOT6: A Novel Regulator of DNA Mismatch Repair

**DOI:** 10.3390/cells11030521

**Published:** 2022-02-02

**Authors:** Peng Song, Shaojun Liu, Dekang Liu, Guido Keijzers, Daniela Bakula, Shunlei Duan, Niels de Wind, Zilu Ye, Sergey Y. Vakhrushev, Morten Scheibye-Knudsen, Lene Juel Rasmussen

**Affiliations:** 1 Center for Healthy Aging, University of Copenhagen, Copenhagen, DK-2200 Copenhagen, Denmark; peng.song@outlook.com (P.S.); 1155152004@link.cuhk.edu.hk (S.L.); guido@sund.ku.dk (G.K.); bakula@sund.ku.dk (D.B.); shunlei.duan@bric.ku.dk (S.D.); mscheibye@sund.ku.dk (M.S.-K.); 2 Department of Cellular and Molecular Medicine, University of Copenhagen, Copenhagen, DK-2200 Copenhagen, Denmark; 3 Department of Spine Surgery, Affiliated Zhongda Hospital, School of Medicine, Southeast University, Nanjing 210009, China; 4 Department of Human Anatomy and Histology, School of Medicine and Holistic Integrative Medicine, Nanjing University of Chinese Medicine, Nanjing 210023, China; liunightelf@aliyun.com; 5 Department of Toxicogenetics, Leiden University Medical Center, 9600, 2300 RC Leiden, The Netherlands; n.de_wind@lumc.nl; 6 Copenhagen Center for Glycomics, University of Copenhagen, Copenhagen, DK-2200 Copenhagen, Denmark; zilu.ye@cpr.ku.dk (Z.Y.); seva@sund.ku.dk (S.Y.V.)

**Keywords:** genome stability, cancer, mismatch repair, mammalian deadenylase, mRNA degradation, gene regulation

## Abstract

DNA mismatch repair (MMR) is a highly conserved pathway that corrects both base–base mispairs and insertion-deletion loops (IDLs) generated during DNA replication. Defects in MMR have been linked to carcinogenesis and drug resistance. However, the regulation of MMR is poorly understood. Interestingly, CNOT6 is one of four deadenylase subunits in the conserved CCR4-NOT complex and it targets poly(A) tails of mRNAs for degradation. CNOT6 is overexpressed in acute lymphoblastic leukemia (ALL), acute myeloid leukemia (AML) and androgen-independent prostate cancer cells, which suggests that an altered expression of CNOT6 may play a role in tumorigenesis. Here, we report that a depletion of CNOT6 sensitizes human U2OS cells to N-methyl-N′nitro-N-nitrosoguanidine (MNNG) and leads to enhanced apoptosis. We also demonstrate that the depletion of CNOT6 upregulates MMR and decreases the mutation frequency in MMR-proficient cells. Furthermore, the depletion of CNOT6 increases the stability of mRNA transcripts from MMR genes, leading to the increased expression of MMR proteins. Our work provides insight into a novel CNOT6-dependent mechanism for regulating MMR.

## 1. Introduction

Genomic stability is critical to maintain normal cell function, and genome maintenance pathways continually respond to environmental and endogenous challenges and various types of cellular stress. DNA mismatch repair (MMR) is a highly conserved pathway that corrects both base–base mispairs and insertion-deletion loops (IDLs) generated during DNA replication. MMR increases the fidelity of DNA replication by several orders of magnitude [1]. MMR is carried out in four main steps, i.e., mismatch recognition, excision, DNA resynthesis and ligation. In human cells, MutSα (MSH2-MSH6) recognizes base-base mismatches and small insertions and deletions (IDLs), while MutSβ (MSH2-MSH3) preferably recognizes larger IDLs [2]. The MutS complex is regulated by ATP binding and hydrolysis, which play vital roles in mismatch recognition [3]. After lesion recognition, the MutLα (MLH1-PMS2) forms a tetrameric complex and converts to a sliding clamp [4]. MMR discriminates the template DNA strand from the nascent DNA strand under the guidance of PCNA, which is required for strand discrimination in human cells [5]. In the next step of MMR, EXO1-dependent or EXO1-independent DNA excision removes mispaired bases on the nascent DNA strand [6]. Then, DNA is resynthesized by high fidelity DNA polymerase (Pol) δ and the nick is ligated by DNA ligase I.

Some MMR proteins play roles in other DNA repair pathways, including base excision repair (BER) [7], double-strand break (DSB) repair [8], nucleotide excision repair (NER) [9] and the Fanconi anaemia (FA) pathway [10]. In addition, MMR is a critical player in the suppression of recombination suppression [11]. MMR influences both the frequency and the outcome of homologous recombination (HR). The divergence of sequences can decrease the recombination rates several-fold [12,13,14]. Together, these studies suggest the considerable contribution of MMR to genome stability.

Dysfunctional MMR increases resistance to certain drugs by up to 100-fold [15]. Furthermore, defects in MMR cause hereditary non-polyposis colon cancer (HNPCC), also named Lynch syndrome (LS), which is the most common form of hereditary colon cancer. Germline mutations in MMR genes *MLH1*, *MSH2*, *MSH6*, and *PMS2* have been reported in HNPCC/LS [16,17]. These mutations significantly increase the risk of colorectal carcinoma (CRC), endometrial carcinoma (EC), and several other cancers [18]. MMR also plays a major role in activating DNA-damage signaling pathways [19].

Microsatellite instability (MSI) is a mutator phenotype characterized by frequent mutations in short repetitive DNA sequences known as microsatellites. MSI was first linked to defects in MMR genes in HNPCC. Although eukaryotic/mammalian MMR has been studied for several decades, novel factors that contribute to MMR continue to emerge, for example MED1 [20], MRE11 [21] and MCM9 [22]. This indicates that our understanding of MMR in mammalian cells may still be limited.

The CCR4-NOT complex is highly conserved from yeast to human cells and localizes to the nucleus and cytoplasm [23]. The main enzymatic functions linked to the CCR4-NOT complex are ubiquitylation and deadenylation [24]. In yeast, the NOT4 subunit (in human named CNOT4) is one of the major players in ubiquitylation, and functions as a RING E3 ligase, which binds to the COOH-terminus of NOT1 to form a stable subunit of the CCR4-NOT complex. Deadenylation by the CCR4-NOT complex is regulated by CCR4 and CAF1 subunits. The CCR4 subunit belongs to the exonuclease–endonuclease-phosphatase (EEP) protein family [25]. CCR4 physically interacts with CAF1 through its leucine-rich repeat (LRR) domain, which docks onto NOT1. They act in the second phase of deadenylation, during which the 3′ poly(A) tails of mRNAs are shortened by up to 20 adenines [26]. Furthermore, CCR4 is thought to play a more critical role during deadenylation than CAF1 [27]. For example, in vitro, CCR4 activity is independent of CAF1 and is retained when CCR4 is used as a monomer in solution [28]. As is known, CCR4 contributes to the DNA damage response (DDR), and the inactivation of CCR4 makes yeast more tolerant to hydroxyurea (HU) and methyl methanesulfonate (MMS) [29]. The mammalian homologs of the CCR4a and CCR4b subunits are CNOT6 and CNOT6L. It is reported that CNOT6 plays a role in DDR [30]. Moreover, CNOT6 and CNOT6L protect cells from senescence and cell death [31]. The altered expression of CNOT6 or CNOT6L has been detected in acute leukemia and androgen-independent prostate cancer cells [32], and single-nucleotide polymorphisms (SNPs) in *CNOT6* have been associated with pediatric acute lymphoblastic leukemia and lung cancer [33].

In order to identify new factors involved in MMR, we conducted a screen for novel MMR regulatory components. One of the proteins we identified to affect MMR expression was CNOT6. In this study, we investigate the molecular mechanisms by which CNOT6 negatively regulates MMR activity. In addition, we show that CNOT6 plays a role in sensitivity to and induction of apoptosis by *N*-methyl-*N*′nitro-*N*-nitrosoguanidine (MNNG). Moreover, our results show that depletion of CNOT6 significantly lowers the mutation frequency in MMR-proficient chromosome 3-complemented HCT116 cells and increases genomic stability in MMR-proficient U2OS cells. Furthermore, we unravel the requirement for CNOT6 deadenylase activity in regulating MMR in the absence of a strong physical interaction between CNOT6 and MMR proteins. Thus, this study provides a significant new insight into the regulation of MMR in human cells.

## 2. Materials and Methods

### 2.1. Cell Culture

Human U2OS osteosarcoma cells were cultured in DMEM medium supplemented with 10% fetal bovine serum (FBS) and 1% penicillin-streptomycin. Human colorectal cancer cells HCT116 (MMR-deficient) and HCT116 + Chr3 (MMR-proficient) were grown in McCoy’s 5A (Modified) Medium with 10% FBS and 1% penicillin-streptomycin. Unless specified, all cells were maintained in a humidified atmosphere of 5% CO_2_ at 37 °C.

### 2.2. High-Throughput Screening

The rationale behind the screening is based on the fact that the sensitivity of the cells to MNNG requires a functional MMR system and that the depletion of MMR proteins such as MSH2, MLH1 and EXO1, promote resistance to killing by MNNG [34]. So, MMR integrity can be reflected by the percentage of live cells after the treatment of MNNG. Human U2OS cells were subjected to roboticized reverse transfection using Lipofectamine RNAiMAX reagent (Thermo Fisher Scientific, Waltham, MA, USA) according to the manufacturer’s protocol in 384-well microplates in a liquid handling station (Hamilton, Bonaduz, GR, Switzerland). The custom-made SilencerSelect libraries of human nucleases from Ambion were used in this assay. After 3-day incubation with siRNA, the cells were treated with 10 µM O^6^-Benzylguanine (O^6^-Bz), an inhibitor of the O^6^-methylguanine DNA methyltransferase (MGMT), or mock-treated with ethanol for 1 h prior to treatment with 200 nM MNNG or mock treatment with DMSO. After three days, the cells were fixed and stained with CytoCalcein TM Green (Bioquest, Zug, Switzerland) for live cells as well as DAPI for nuclear DNA. The relative percentage of live cells was calculated as (number of GFP-positive cells in drug-treated wells)/(GFP-positive cells in parallel mock-treated wells) and the relative cell number was calculated as (DAPI signal intensity in drug treated wells)/(DAPI signal intensity in parallel mock treated wells). Cells transfected with siMLH1 were used as the positive control for MMR deficiency and siLUC as a positive control for MMR proficiency. Two independent experiments were performed, and data were analyzed using Fisher’s combined probability test. Candidate genes were selected that either (1) showed increased resistance to MNNG treatment indicating decreased MMR activity or (2) decreased resistance to MNNG treatment indicating increased MMR activity.

### 2.3. siRNA Sequences, DNA Constructs and Antibodies

The siRNA targeting different genes are listed below: 5′-CGUACGCGGAAUACUUCGAUU-3′ for control (siLUC), and 5′-CUUGAGGAGUUUCAGUAUA-3′ for *MSH2* (siMSH2). The siRNAs targeting at *CNOT6*, *CNOT6L*, *CNOT7* and *CNOT8* are the same as those used in previous publications [35]. The pcDNA3.1+/C-(K)DYK-*CNOT6* plasmid used to overexpress Flag-tag fused CNOT6 protein was purchased from GenScript (New Jersey, NJ, USA), as well as the vector pcDNA3.1+/C-(K)DYK. pT7-EGFP-C1-Hs*Not6* was a gift from Elisa Izaurralde (37368, Addgene, Watertown, MA, USA) [36]. All plasmids were validated by sequencing. The mismatched DNA substrate for the in vitro MMR assay has been reported before [37]. Primary antibodies were MSH2 (NA27, Sigma-Aldrich, Darmstadt, Germany), CNOT6 (13415S, Cell Signalling, Beverly, MA, USA), CNOT7 (101009, Santa Cruz, Heidelberg, Germany), Actin (RF2228263, Thermo Fisher Scientific), MSH6 (sc-1242, Santa Cruz), MSH3 (611390, BD Pharmingen, New Jersey, NJ, USA), MLH1 (554073, BD Pharmingen), PMS2 (556415, BD Pharmingen), GFP (11814460001, Roche, Basel, Switzerland), RFC1 (ab3566, Abcam, Waltham, MA, USA), PCNA (sc-56, Santa Cruz) and γH2AX (05636, Sigma-Aldrich). The secondary antibodies were goat anti-rabbit IgG (ab205718, Abcam), goat anti-mouse IgG (G-21040, Thermo Fisher Scientific), mouse anti-goat IgG (sc-2354, Santa Cruz), and Alexa Fluor 568 goat anti-mouse IgG (A11004, Thermo Fisher Scientific).

### 2.4. Cell Transfection

Cells were transfected for 24 h after seeding using Lipofectamine™ RNAiMAX Transfection Reagent (13778075, Thermo Fisher Scientific) following the manufacturer’s protocol. The growth medium was changed 24 h post-transfection. For overexpression, plasmids were transfected into cells using PolyJet (SL100688, Tebu-bio, Roskilde, Denmark) according to the manufacturer’s protocol. Cells were maintained for 24 h before the growth medium was changed.

### 2.5. Protein Immunoblotting and Immunoprecipitation

Total protein was extracted 72 h after siRNA transfection or 48 h after plasmid transfection using RIPA buffer (89901, Thermo Fisher Scientific) supplemented with protease inhibitor (4693159001, Sigma-Aldrich). Cell lysates were centrifuged at 20,000× *g* at 4 °C for 20 min to remove cell debris. Protein concentration was determined using a BCA assay kit (23227, Thermo Fisher Scientific). Equal amount of protein was loaded and separated on 4–12% Bis-Tris gels. After membrane transfer, the membrane was incubated with primary antibodies overnight at 4 °C. Then, secondary antibodies were applied, and ECL Substrate (1705061, Bio-Rad, Copenhagen, Denamrk) was used to detect the bands under the ChemiDoc Imaging developer (Bio-Rad). Immunoprecipitation was performed as described [38]. In brief, cells transfected with plasmids were lysed in an IP lysis buffer (25 mM Tris-HCl pH 8.0, 150 mM NaCl, 1% IGEPAL, 1 mM EDTA, 2 mM MgCl_2_) supplemented with protease inhibitor and Benzonase (sc-202391, Santa Cruz). After removing cell debris, the lysates were incubated with GFP-TRAP beads (gtma-20, ChromoTek, Munich, Germany) overnight at 4 °C. The beads were washed four times in a washing buffer (25 mM Tris-HCl pH 8.0, 150 mM NaCl, 1% IGEPAL, 1 mM EDTA) supplemented with protease inhibitor. The beads were resuspended in a 1× Laemmli sample buffer and boiled at 95 °C for 7 min. Samples were separated on 4–12% Bis-Tris gels.

### 2.6. RNA Isolation and Quantitative Real-Time PCR

Total RNA was isolated at the indicated time points using RNeasy Plus Mini Kit (74134, Qiagen, Hilden, Germany). cDNA was prepared with 1 μg total RNA by High-Capacity cDNA Reverse Transcription Kit (4368814, Thermo Fisher Scientific). The reaction was diluted 20 times. Quantitative real-time PCR was performed according to manufacturer’s protocol (08-36-00001, TAG Copenhagen, Denmark) using an RTq-PCR machine (Thermo Fisher Scientific). Primers were designed using https://blast.ncbi.nlm.nih.gov/Blast.cgi accessed on 28 July 2018 and sequences can be obtained upon request.

### 2.7. Cell Viability (MTS) Assay

Cells were seeded into 96-well plates. Then, 24 h later, cells were transfected with siRNAs or plasmids. 72 or 48 h after transfection, cells were incubated with 10 μM O^6^-Bz for 1 h before different concentrations of MNNG were added to the wells. Cells were incubated with MNNG for 1 h followed by the change of fresh medium containing 10 μM O^6^-Bz. Cells were maintained for 72 h. Then, MTS reagent (G3580, Promega, Täby, Sweden) was added to each well and incubated for 3 h at 37 °C. Absorbance at 490 nm was measured on a 96-well plate reader after shaking.

### 2.8. Apoptosis Detection by Flow Cytometry

In the 24 h after seeding, cells were transfected with siRNAs. 72 h after transfection, cells were incubated with 10 μM O^6^-Bz for 1 h before 0.5 μM MNNG were added to the wells. After 1 h of incubation, MNNG was removed and followed by addition of fresh growth medium containing 10 μM O^6^-Bz. Cells were maintained for 72 h. Apoptosis was measured according to manufacturer’s protocol (ab14153, Abcam). In brief, 0.8 × 10^5^ cells were resuspended in 250 μL 1× Binding Buffer with the addition of 2.5 µL of Annexin V-EGFP and incubated for 15 min at room temperature. Quantification of apoptosis was performed by flow cytometry using a CytoFLEX flow cytometer (Beckman Coulter, Inc., Brea, CA, USA).

### 2.9. mRNA Stability Assay

An mRNA stability assay was performed as described previously [39]. In the 72 h after transfection, Actinomycin D, which is a transcriptional inhibitor, was added to the growth medium at a final concentration of 5 µg/mL. Total RNA was isolated 0, 3, 6 and 12 h after the treatment. Reverse transcription, RTq-PCR was carried out to determine the mRNA expression levels of target genes. *GAPDH* was used as the reference gene. mRNA half-lives were calculated as described [39].

### 2.10. HPRT Mutation Assay

The HPRT mutation assay was carried out to determine the mutation frequencies under various conditions as described previously [40]. In brief, both MMR-deficient HCT116 and MMR-proficient HCT116 + Chr3 cells were maintained in growth medium containing 1× hypoxanthine–aminopterin–thymidine (HAT) for 3 days followed by incubation for 24 h in growth medium containing 1× hypoxanthine–thymidine (HT). Then, both cell lines were transfected with siLUC or siCNOT6 three times every third day. Three days post the third transfection cell were counted. For HCT116, 200 cells were plated in each well in triplicates on 6-well plate in the medium without the purine analogue 6-thioguanine (6-TG). Moreover, 200,000 cells were plated in the same way, but in the medium with 6-TG at the final concentration of 0.6 µg/mL. For HCT116 + Chr3, 200 cells were plated in each well in triplicates on 6-well plate in the medium without 6-TG. Then, the remaining cells were split into two 15 cm dishes in the medium with 0.6 µg/mL 6-TG. Cells were incubated for at least nine days before crystal violet staining. Colonies were counted. Mutation frequency was calculated as the ratio of the cloning efficiency with 6-TG to the cloning efficiency without 6-TG.

### 2.11. In Vitro MMR Assay

The in vitro MMR assay was performed as described previously [41]. 72 h after transfection, nuclear extracts were prepared following manufacturer’s protocol of CelLytic™ NuCLEAR™ Extraction Kit (NXTRACT-1KT, Sigma-Aldrich). One hundred nanogram of G/T substrate was incubated at 37 °C for 45 min with 75 µg nuclear extracts in 35 µL reactions. The reactions were terminated by the addition of stop solution (50 mM EDTA, 2% SDS, and 2 mg/mL proteinase K). The mixture was further incubated at 37 °C for 45 min. The following steps were applied as described [42]. In brief, restriction digestions were carried out in 12 µL reactions after DNA purification containing *Nla*III endonuclease (R0125S, NEB, Ipswich, MA, USA) for 60 min at 37 °C. The digested substrate was mixed with Hi-Di Formamide containing GeneScan™ 500 ROX™ dye (401734, Thermo Fisher Scientific) and fragment analysis was performed on a 3130 DNA analyzer (Thermo Fisher Scientific). Data were analyzed using GeneMarker V1.5 software (State College, PA, USA). Then, the repair levels were calculated by dividing the height of the MMR-specific peak by the total fluorescent signal.

## 3. Results

### 3.1. Depletion of the Deadenylase Subunit of the CCR4-NOT Complex Sensitizes U2OS Cells to MNNG

**Figure 1:**
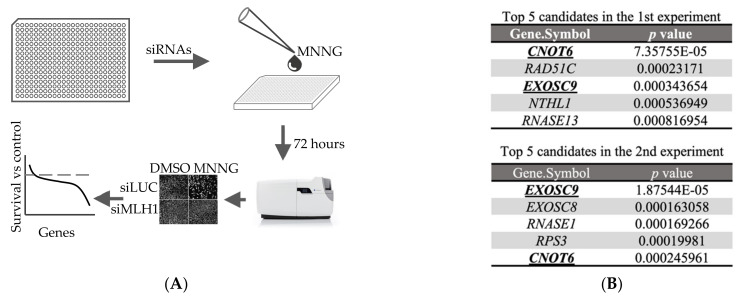

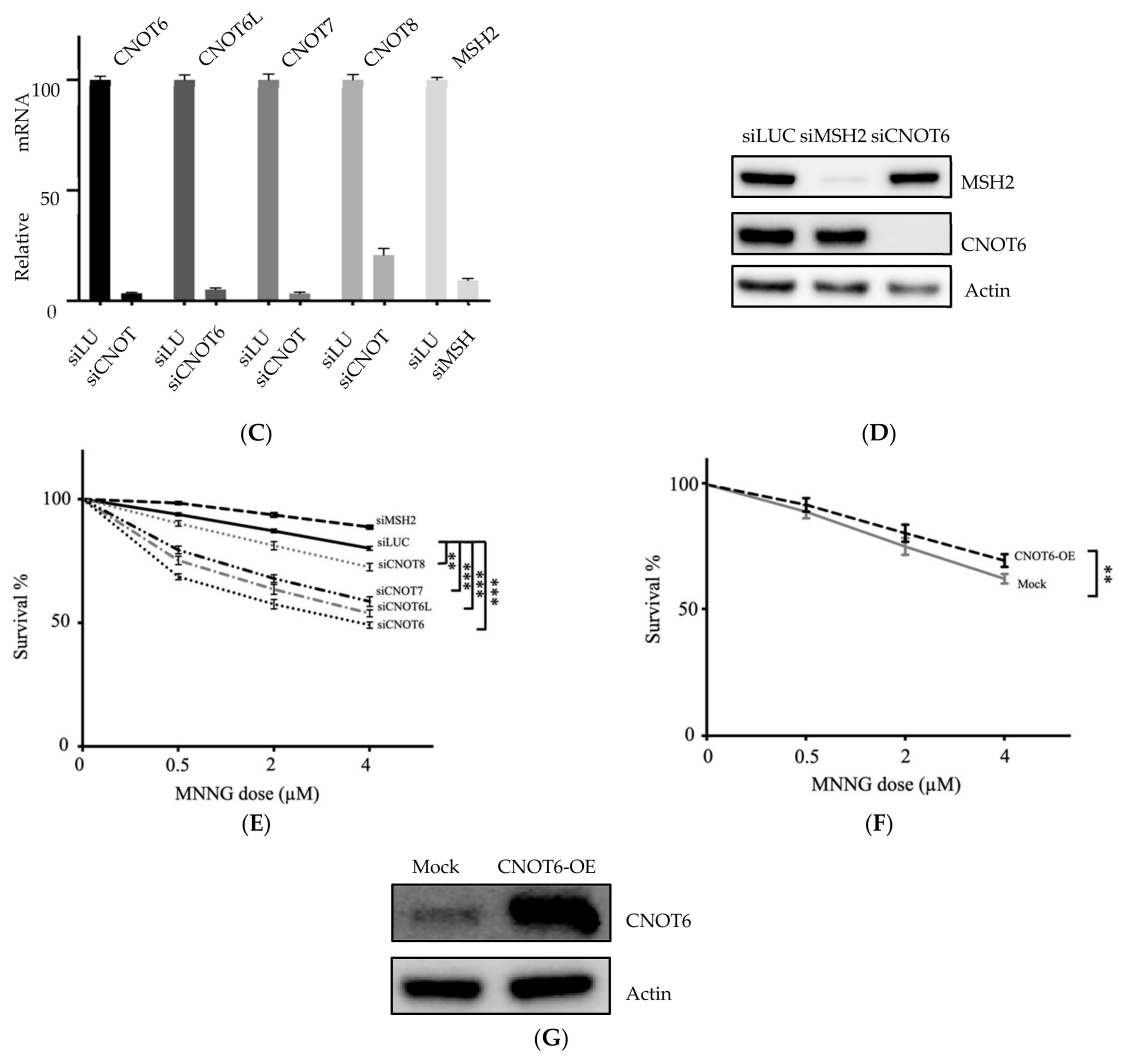
siRNA-based high-throughput screen identifies CNOT6 as a potential MMR regulator. (**A**) Schematic depiction of the screening procedure combining custom made siRNA libraries of 160 human nucleases, MNNG treatment. The screening endpoint was cell survival. (**B**) The top five candidates from two independent experiments are shown. (**C**) SiRNA-mediated knockdown efficiency was quantified for the indicated genes by real-time quantitative PCR. *GAPDH* was the reference gene (*n* = 3). (**D**) Representative immunoblots of MSH2, CNOT6 and Actin in extracts of U2OS cells treated with siRNA targeting CNOT6 or MSH2. (**E**) The viability of siRNA- transfected cells treated with MNNG at the indicated dose is shown. Data are shown as mean ± SEM, *n* = 3. Statistical significance (** *p* < 0.01, *** *p* < 0.001) was determined using an unpaired two-tailed Student’s *t* test. (**F**) Cell viability in the presence of the indicated dose of MNNG is shown; relative survival is shown for cells overexpressing CNOT6 or control cells. Data are shown as mean ± SD, *n* = 3, ** *p* < 0.01, using unpaired two-tailed Student’s *t* test. (**G**) Representative immunoblots of CNOT6 and Actin in extracts of U2OS cells overexpressing CNOT6 or control cells.

In order to identify new components of MMR, we used siRNA-mediated high-throughput screening to identify novel factors involved in human MMR (Figure 1A). The screen identified the known MMR factor MLH1 as well as other putative positives. The top five candidates in two independent experiments are shown in Figure 1B; the two top-scoring hits were EXOSC9 and CNOT6. EXOSC9 is linked to the rare disorder pontocerebellar hypoplasia [42], the expression of CNOT6 is reported to be altered in several types of tumor cells, and it has been suggested that it plays a role in preventing cell death and senescence [43]. Therefore, we chose to investigate CNOT6 further.

To validate CNOT6 as a positive hit and putative regulator of human MMR, we measured the viability of CNOT6-depleted human O2OS cells in the presence and absence of MNNG. The human CCR4-NOT complex consists of four deadenylase subunits, CNOT6, CNOT6L, CNOT7 and CNOT8. To gain insight into the role of this complex in the regulation of MMR, siRNA was used to selectively knockdown each of the four deadenylase subunits in U2OS cells (Figure 1C,D). The depletion of each subunit made the cells sensitive to MNNG (Figure 1E). Furthermore, the CNOT6-, CNOT6L- and CNOT7-depleted cells were more sensitive to killing by MNNG than cells depleted of CNOT8, and CNOT6-depleted cells had the most profound increase in sensitivity to MNNG. To further verify this observation, CNOT6 was overexpressed in U2OS cells, which were then challenged with MNNG. The results show that overexpression of CNOT6 in U2OS cells increase resistance to MNNG, while overexpression of empty vector did not (Figure 1F,G), which is consistent with decrease in MMR activity. No significant difference was observed between the control and CNOT6-depleted MMR-deficient HCT116 cells after MNNG treatment (data not shown). Together, these results suggest that CNOT6 plays a specific role in the response to MNNG-induced cellular damage, which is consistent with a role for CNOT6 as a negative regulator of MMR.

### 3.2. Knockdown of CNOT6 Increases the Frequency of MNNG-Induced Apoptosis

**Figure 2:**
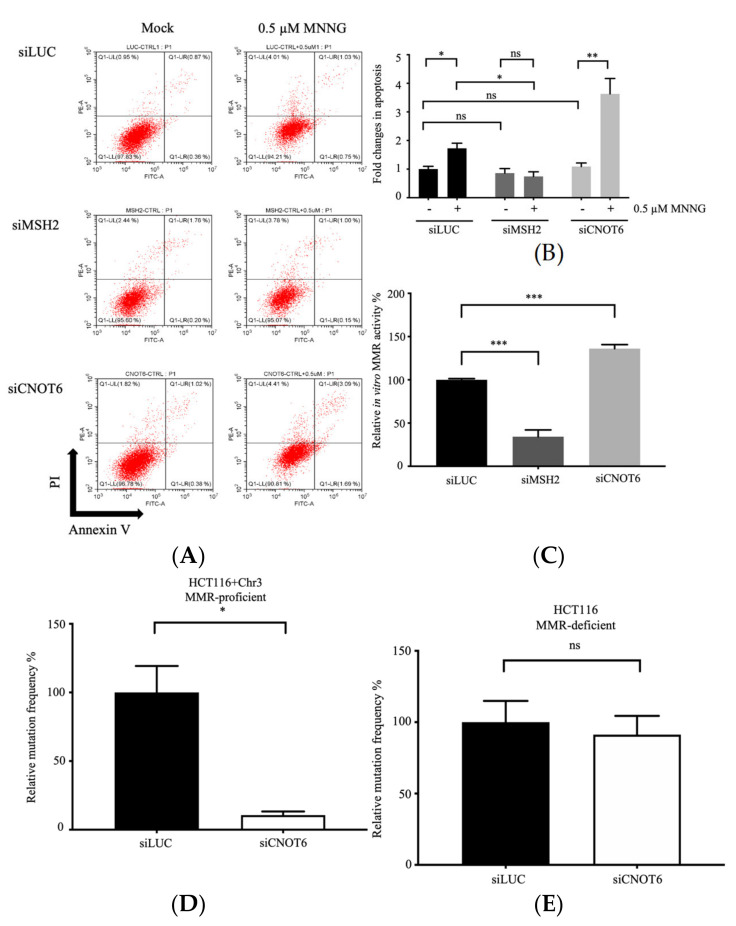
Depletion of CNOT6 increases apoptosis after exposure to MNNG, stimulates MMR and reduces mutation frequency in MMR-proficient cells. (**A**) Representative scatter plots of PI vs. Annexin V staining of U2OS cells transfected with siRNA targeting LUC, MSH2 or CNOT6 and treated with or without 0.5 μM MNNG as described in Materials and Methods. (**B**) Quantification of flow cytometry data. Data are shown as mean ± SEM, *n* = 3; “ns” signifies “not significant”, * *p* < 0.5, ** *p* < 0.01, using unpaired two-tailed Student’s *t* test. (**C**) MMR activity in extracts from MSH2-depleted or CNOT6-depleted or control siLUC-treated U2OS cells. MMR was quantified using in vitro MMR assay (see Materials and Methods). Data are shown as mean ± SEM, *n* = 3; Statistical significance (*** *p* < 0.001) was determined using unpaired two-tailed Student’s *t* test. (**D**,**E**) *HPRT* assay was used to estimate mutation frequency in CNOT6-depleted (**D**) HCT116 + Chr3 (MMR-proficient) and (**E**) HCT116 (MMR-deficient) cells. Data are shown as mean ± SEM, *n* = 3; statistical significance (* *p* < 0.5) was determined using unpaired two-tailed Student’s *t* test.

MMR activates DNA damage signaling pathways [16,40,41,42], which in turn can increase cell death by apoptosis. To examine whether the depletion of CNOT6 increases apoptosis, MNNG-treated and control mock-treated U2OS cells with or without depletion of CNOT6 were stained with propidium iodide (PI) and Annexin V and analyzed by flow cytometry. Untreated control and CNOT6-depleted cells had similar levels of apoptosis (Figure 2A,B). Although both control and CNOT6-depleted cells showed a significant increase in apoptosis after treatment with MNNG treatment, the increase was more than two-fold higher in CNOT6-depleted cells than in control cells (3.6-fold vs. 1.7-fold). Furthermore, a similar increase was not observed in MSH2-deficient cells. These results show that the depletion of CNOT6 decreases cell viability in the presence of MNNG because it leads to an increase in MNNG-induced apoptosis. This increased apoptosis is caused by increased DSBs measured as γH2AX foci by high-content microscopy in MNNG treated CNOT6-depleted cells (Figure S1).

### 3.3. CNOT6 Regulates MMR Activity In Vitro

MNNG is an S_N_1-type methylating agent that modifies purine nitrogen atoms, causing potentially mutagenic DNA lesions. MNNG introduces O^6^-methylguanine (^O6Me^G) DNA lesions into DNA, which are repaired by MGMT. Although typically, most modified purines are removed from DNA, ^O6Me^G can persist when MGMT is inactivated, for example, by exposure to O^6^-Bz. Previous studies show that MMR-deficient cells can be up to 100-fold more resistant to S_N_1-type methylating agents than the corresponding MMR-proficient cells [15]. This MNNG resistant phenotype in MMR-deficient cells exposed to MNNG is caused by the absence of ^O6Me^G:T mismatches being recognized by MMR leading to less DSBs and a lower rate of cell death by apoptosis. Because CNOT6-depletion sensitizes U2OS cells to MNNG, we hypothesized that CNOT6 negatively regulates MMR in these cells. To validate this hypothesis, extracts were prepared from CNOT6-depleted, MSH2-depleted and control cells and their relative MMR activity on a G/T mismatch DNA substrate was quantified using an in vitro MMR assay. As shown in Figure 2C, MMR activity was significantly lower in MSH2-depleted cells than in control cells, but it was significantly higher in CNOT6-depleted cells than in control cells. This result is consistent with the hypothesis that CNOT6 represses MMR activity.

### 3.4. Depletion of CNOT6 Decreases Mutation Frequency in MMR-Proficient Cells, but Not in MMR-Deficient Cells

Because CNOT6 is implicated to be a repressor of MMR, the effect of CNOT6 depletion on in vivo mutation frequency was investigated in MMR-deficient HCT116 and MMR-proficient HCT116 + Chr3 (chromosome 3-complemented) cells using the hypoxanthine phosphoribosyl transferase (HPRT) mutation assay. The human *HPRT* gene is located on the X chromosome and the inactivation of HPRT leads to resistance to 6-TG; in the HPRT assay, mutation frequency is estimated by measuring sensitivity/resistance to 6-TG. To ensure that the predicted change in mutation frequency was sufficiently large to be observed, cells were treated with CNOT6 siRNA or control siLUC siRNA three times. Notably, the results showed that mutation frequency was significantly higher in MMR-proficient HCT116 + Chr3 cells (9.86 × 10^−6^) than in CNOT6-depleted (1.05 × 10^−6^) HCT116 + Chr3 cells (Figure 2D), while the mutation frequencies were similar in MMR-deficient HCT116 cells (4.27 × 10^−4^) and CNOT6-depleted (3.90 × 10^−4^) HCT116 cells (Figure 2E). These results demonstrate that the effect of CNOT6 on mutation frequency requires functional MMR. Interestingly, the mutation frequency of CNOT6-depleted MMR-proficient cells was ~10-fold lower compared to the mock-depleted MMR-proficient cells. These results confirm that CNOT6 negatively regulates MMR thereby influencing genome stability but whether the effect of CNOT6 is specific for the MMR pathway, or the effect is rather consequence of an overall (bulk) increased mRNA stability is unknown. However, since we did not observe a reduction in mutation frequency in MMR-deficient HCT116 cells depleted for CNOT6, the results suggest that DNA repair pathways other than MMR play a minor role in mutation avoidance in CNOT6-depleted cells.

### 3.5. Absence of Physical Interaction between CNOT6 and MMR Proteins

**Figure 3:**
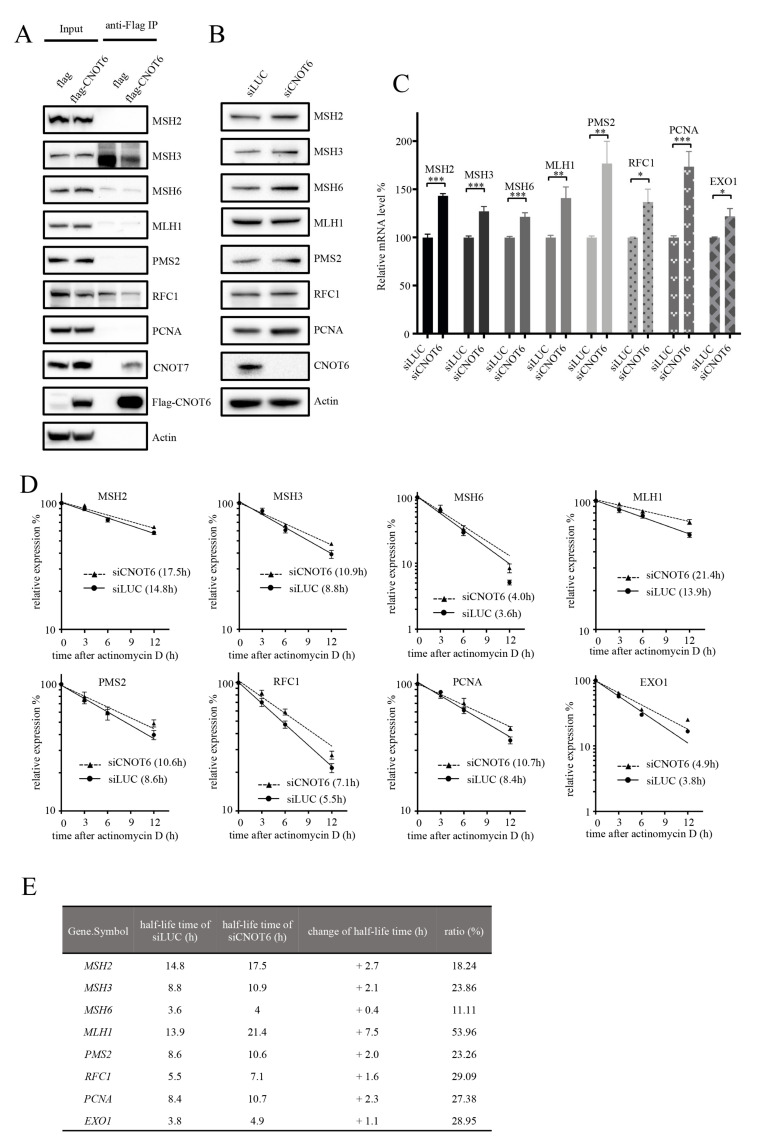
CNOT6 deficiency stabilizes MMR mRNA transcripts. (**A**) U2OS cells transiently overexpressing Flag-tagged-CNOT6 were collected and lysed, and cell extracts used for anti-Flag immunoprecipitation. Representative immunoblots for the indicated proteins are shown. (**B**) Representative immunoblots for the indicated MMR factors using extracts from CNOT6-depleted cells. (**C**) Real-time quantitative PCR was performed to quantify expression of the indicated MMR genes in CNOT6-depleted cells. Data are shown as mean ± SEM, *n* = 3; statistical significance (* *p* < 0.5, ** *p* < 0.01, *** *p* < 0.001) was determined using unpaired two-tailed Student’s *t* test. (**D**) The half-life of MMR gene transcripts in control and CNOT6-depleted cells with the treatment of transcriptional inhibitor Actinomycin D is shown. *n* = 3. (**E**) Data from panel D are compared to demonstrate the effect of CNOT6-depletion on the half-life of MMR gene transcripts.

As MMR activity increased and mutation frequency decreased in CNOT6-depleted cells, we investigated whether CNOT6 directly interacts with MMR proteins using Flag-tagged CNOT6 as “bait”. We did not detect physical interaction between MMR proteins and CNOT6 complexes (Figure 3A). Notably, the previously characterized CNOT6 interaction partner CNOT7 was identified (Figure 3A). Furthermore, a mass spectrometry-based approach also failed to identify interactions between CNOT6 and MMR proteins (Text S1). Nevertheless, because these are negative results, we cannot completely rule out the possibility that CNOT6 does physically interact with one or more MMR proteins under certain circumstances in vivo.

### 3.6. Knockdown of CNOT6 Stabilizes mRNA Transcripts through Decreased Deadenylation

Our results suggest that the increased MMR activity in CNOT6-depleted cells does not require physical interaction between CNOT6 and MMR proteins. Another possibility is that CNOT6 regulates the expression of MMR proteins. Consistent with this idea, mRNA and protein products of MMR genes were detected at higher levels in CNOT6-depleted cells than in the control cells (Figure 3B,C).

Acting as the deadenylase subunit of the CCR4-NOT complex, CNOT6 removes 3′ poly(A) tails from mRNAs, leading to mRNA decapping and degradation [26]. It has been reported that CNOT6 displays deadenylase activity both in vitro and in vivo [28]. Therefore, depletion of CNOT6 could deplete or reduce mRNA deadenylation, which would stabilize mRNAs. To test this hypothesis, mRNA stability and abundance was quantified in the presence of Actinomycin D, which inhibits transcription, in CNOT6-depleted and control cells. Notably, we observed that the half-life of *MLH1* mRNA increased 53.96%, while the half-life of *MSH6* mRNA increased by 11.11% (Figure 3D,E). These results confirm that depletion of CNOT6 increases the stability and the abundance of MMR mRNA. This was further confirmed by measuring the poly(A) tail-lengths of *MSH2* and *MLH1* at 0 or 12 h after the addition of Actinomycin D (Figure S2). Overexpression of CNOT6 did not specifically affect the stability of *CSB*, *ATR*, and *RAD51* mRNAs (Figure S3). This result shows that deadenylation by CNOT6 regulates MMR mRNA stability.

## 4. Discussion

MMR is highly conserved among prokaryotic and eukaryotic species. Although dysfunctional MMR has been linked to tumorigenesis and to drug resistance [43,44], the regulation of MMR remains poorly understood. Here, we report that depletion of the CNOT6 subunit of the CCR4-NOT complex increases the sensitivity of U2OS cells to MNNG and upregulates MMR. The ^O6Me^G lesions generated by MNNG are mutagenic by a mechanism involving mis-incorporation of thymine opposite the ^O6Me^G adduct. The resulting ^O6Me^G:T mismatch can lead to a G:C to A:T transition mutation if the DNA lesion is not repaired before the completion of DNA replication and cell division [43]. In the absence of MGMT, the unrepaired ^O6Me^G:T mispair is recognized by MutSα, and the MMR machinery enters a so-called “futile cycle”, in which thymine is repeatedly excised and mis-incorporated opposite ^O6Me^G. When a nick or gap generated during MMR is not directly repaired, it can also be converted into a DSB [44], which activates a G2 checkpoint and subsequent cell-cycle arrest [45]. Therefore, higher MMR activity correlates with greater sensitivity to MNNG in MGMT inactivated cells. This is consistent with our observations that CNOT6-depleted cells demonstrate increased sensitivity to MNNG (Figure 1E), and enhanced MMR activity (Figure 2C).

Both human CNOT6 and its yeast ortholog CCR4 possess 3′-5′ poly(A) exoribonuclease activity [28]. In CCR4-deficient yeast, poly(A) tails of ~30 adenosines accumulate, while mRNA species with poly(A) tails shorter than ~22 adenosines reduce markedly; in contrast, in the control wild-type strain, poly(A) tails are shortened to ~10 adenosines [46]. This suggests a defect in the deadenylation in the CCR4-deficient strain. The length of poly(A) tails is critical for mRNA stability, because it protects mRNA from 3′ degradation, decapping and 5′ mRNA decay [47]. The results presented here demonstrate that CNOT6/CCR4 stimulates mRNA turnover through its deadenylase activity. In line with this, we show that depletion of CNOT6 stabilizes MMR mRNAs (Figure 3C,D) and increases the expression of MMR proteins (Figure 3B). It would be interesting to investigate the role of CNOT6 on a larger panel of other genes. However, due to the main aim of this study is at the regulation of MMR genes, we chose to focus on MMR and only included few BER and DSB repair genes as control for the specificity of CNOT6 on MMR gene stability. Our results do not clearly show if CNOT6 also affect the stability of other repair mRNAs. Therefore, we cannot exclude that some of the phenotypes observed are caused by deregulation of DNA repair pathways other than MMR.

In addition to its role in DNA repair and genome stability, MMR can activate DNA-damage signaling pathways and induce apoptosis, which is a strategy to avoid pathways leading to tumorigenesis [19]. Moreover, apoptosis is a known consequence of chemotherapeutic treatment, and resistance to chemotherapy can be associated with the inability to activate cell death pathways in response to DNA damage. It has been proposed that MSH2 and MSH6 play roles in apoptosis independent of their roles in DNA repair [41,42,48]. An overexpression of MLH1 also leads to enhanced apoptosis in human cells [49]. Interestingly, we observed that the frequency of apoptosis is higher in MNNG-treated CNOT6-depleted cells compared to that in MNNG-treated mock-depleted cells (Figure 2A,B), which agrees with previous studies [41,42,50]. Depletion of CNOT6 in MMR-proficient cells does not lead to increased apoptosis without MNNG treatment (Figure 2A,B). This result could be explained by the fact of the endogenous level of ^O6Me^G is not being high enough to induce a significant number of DSBs.

**Figure 4:**
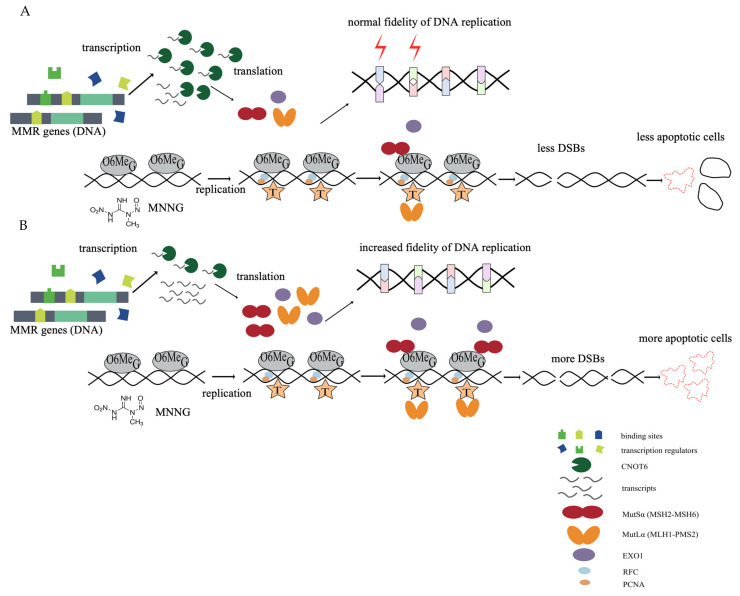
Proposed model for the regulation of MMR by CNOT6. (**A**) The stability of MMR transcripts under normal condition. (**B**) Depletion of CNOT6 leads to enhanced MMR, follow by increased DSBs and apoptosis. To explain the increased DSBs and apoptosis in CNOT6-depleted cells after MNNG treatment, there are several hypotheses. (a) The depletion of CNOT6 leads to unbalanced increase of MMR proteins, due to the different extension of mRNA half-life. (b) Increased MMR proteins may lead to more proteins that bind to the mispairs, which could impede the repair progress. (c) MMR can suppress recombination, which is important in DSB repair. (d) Processing by multiple repair pathways at the same site could slow down or interfere the repair. All these could activate a G2 checkpoint and subsequent cell cycle arrest.

Previous studies on the regulation of MMR report that UV-stimulated transcription of *MSH2* is enhanced by p53 and c-Jun through p53- and AP-1 binding sites in the promoter region upon UV irradiation [50]. Similarly, the *MSH6* promoter is upregulated when Sp1 and Sp3 transcription factors bind the 5′-flanking region [51]. While these studies show that MMR gene promoters interact directly with transcription factors that modulate promoter activity, our results suggest another regulatory pathway involving CNOT6-mediated regulation of mRNA stability through deadenylation (Figure 4). It is also worth noting that some tumors demonstrate decreased expression of CNOT6 [52], while a higher expression of CNOT6 has been reported in acute lymphoblastic leukemia (ALL), acute myeloid leukemia (AML) and androgen-independent prostate cancer [32,53].

## 5. Conclusions

We provided evidence that human MMR is regulated post-transcriptionally by CNOT6, one subunit of the deadenylase activity associated with the CCR4-NOT complex. A depletion of CNOT6 stabilizes mRNA transcripts of MMR genes and increases MMR activity in MMR-proficient U2OS cells. Furthermore, CNOT6 appears to be more important in this regard than the other three deadenylase subunits of the CCR4-NOT complex. To provide more generalizable evidence for the proposed CNOT6 roles, experiments should be repeated in a noncancerous (normal) cell line.

## Data Availability

Not applicable.

## Supplementary Materials

The following are available online at https://www.mdpi.com/article/10.3390/cells11030521/s1, Figure S1: More damages were induced by MNNG treatment in CNOT6-depleted cells through the detection of γH2AX foci formation, Figure S2: The impact of CNOT6 depletion on poly(A) tail-length, Figure S3: Effect of CNOT6 overexpression on various DNA repair mRNA transcripts and Text S1: Mass spectrometry (MS) analysis.

## References

[1] Schofield M.J., Hsieh P. (2003). DNA Mismatch Repair: Molecular Mechanisms and Biological Function. Annu. Rev. Microbiol..

[2] Li G.-M. (2008). Mechanisms and Functions of DNA Mismatch Repair. Cell Res..

[3] Alani E., Lee J.Y., Schofield M.J., Kijas A.W., Hsieh P., Yang W. (2003). Crystal Structure and Biochemical Analysis of the MutS·ADP·Beryllium Fluoride Complex Suggests a Conserved Mechanism for ATP Interactions in Mismatch Repair. J. Biol. Chem..

[4] Qiu R., Sakato M., Sacho E.J., Wilkins H., Zhang X., Modrich P., Hingorani M.M., Erie D.A., Weninger K.R. (2015). MutL Traps MutS at a DNA Mismatch. Proc. Natl. Acad. Sci. USA.

[5] Pluciennik A., Dzantiev L., Iyer R.R., Constantin N., Kadyrov F.A., Modrich P. (2010). PCNA Function in the Activation and Strand Direction of MutL Endonuclease in Mismatch Repair. Proc. Natl. Acad. Sci. USA.

[6] Kadyrov F.A., Holmes S.F., Arana M.E., Lukianova O.A., O’Donnell M., Kunkel T.A., Modrich P. (2007). Saccharomyces Cerevisiae MutLα Is a Mismatch Repair Endonuclease. J. Biol. Chem..

[7] Schanz S., Castor D., Fischer F., Jiricny J. (2009). Interference of Mismatch and Base Excision Repair during the Processing of Adjacent U/G Mispairs May Play a Key Role in Somatic Hypermutation. Proc. Natl. Acad. Sci. USA.

[8] Shahi A., Lee J.-H., Kang Y., Lee S.H., Hyun J.-W., Chang I.-Y., Jun J.-Y., You H.J. (2011). Mismatch-Repair Protein MSH6 Is Associated with Ku70 and Regulates DNA Double-Strand Break Repair. Nucleic Acids Res..

[9] Bertrand P., Tishkoff D.X., Filosi N., Dasgupta R., Kolodner R.D. (1998). Physical Interaction between Components of DNA Mismatch Repair and Nucleotide Excision Repair. Proc. Natl. Acad. Sci. USA.

[10] Peng M., Litman R., Xie J., Sharma S., Brosh R.M., Cantor S.B. (2007). The FANCJ/MutLα Interaction Is Required for Correction of the Cross-Link Response in FA-J Cells. EMBO J..

[11] Spies M., Fishel R. (2015). Mismatch Repair during Homologous and Homeologous Recombination. Cold Spring Harb. Perspect. Biol..

[12] Sugawara N., Goldfarb T., Studamire B., Alani E., Haber J.E. (2004). Heteroduplex Rejection during Single-Strand Annealing Requires Sgs1 Helicase and Mismatch Repair Proteins Msh2 and Msh6 but Not Pms1. Proc. Natl. Acad. Sci. USA.

[13] Heyer W.-D., Ehmsen K.T., Liu J. (2010). Regulation of Homologous Recombination in Eukaryotes. Annu. Rev. Genet..

[14] Welz-Voegele C., Jinks-Robertson S. (2008). Sequence Divergence Impedes Crossover More than Noncrossover Events during Mitotic Gap Repair in Yeast. Genetics.

[15] Karran P. (2001). Mechanisms of Tolerance to DNA Damaging Therapeutic Drugs. Carcinogenesis.

[16] Bronner C.E., Baker S.M., Morrison P.T., Warren G., Smith L.G., Lescoe M.K., Kane M., Earabino C., Lipford J., Lindblom A. (1994). Mutation in the DNA Mismatch Repair Gene Homologue HMLH 1 Is Associated with Hereditary Non-Polyposis Colon Cancer. Nature.

[17] Miyaki M., Konishi M., Tanaka K., Kikuchi-Yanoshita R., Muraoka M., Yasuno M., Igari T., Koike M., Chiba M., Mori T. (1997). Germline Mutation of MSH6 as the Cause of Hereditary Nonpolyposis Colorectal Cancer. Nat. Genet..

[18] Umar A., Boland C.R., Terdiman J.P., Syngal S., de la Chapelle A., Rüschoff J., Fishel R., Lindor N.M., Burgart L.J., Hamelin R. (2004). Revised Bethesda Guidelines for Hereditary Nonpolyposis Colorectal Cancer (Lynch Syndrome) and Microsatellite Instability. J. Natl. Cancer Inst..

[19] Li G.M. (1999). The Role of Mismatch Repair in DNA Damage-Induced Apoptosis. Oncol. Res..

[20] Bellacosa A., Cicchillitti L., Schepis F., Riccio A., Yeung A.T., Matsumoto Y., Golemis E.A., Genuardi M., Neri G. (1999). MED1, a Novel Human Methyl-CpG-Binding Endonuclease, Interacts with DNA Mismatch Repair Protein MLH1. Proc. Natl. Acad. Sci. USA.

[21] Her C., Vo A.T., Wu X. (2002). Evidence for a Direct Association of HMRE11 with the Human Mismatch Repair Protein HMLH1. DNA Repair.

[22] Traver S., Coulombe P., Peiffer I., Hutchins J.R.A., Kitzmann M., Latreille D., Méchali M. (2015). MCM9 Is Required for Mammalian DNA Mismatch Repair. Mol. Cell.

[23] Collart M.A. (2016). The Ccr4-Not Complex Is a Key Regulator of Eukaryotic Gene Expression. Wiley Interdiscip. Rev. RNA.

[24] Collart M.A., Panasenko O.O. (2017). The Ccr4-Not Complex: Architecture and Structural Insights. Subcell Biochem..

[25] Dlakić M. (2000). Functionally Unrelated Signalling Proteins Contain a Fold Similar to Mg2+-Dependent Endonucleases. Trends Biochem. Sci..

[26] Yamashita A., Chang T.-C., Yamashita Y., Zhu W., Zhong Z., Chen C.-Y.A., Shyu A.-B. (2005). Concerted Action of Poly(A) Nucleases and Decapping Enzyme in Mammalian MRNA Turnover. Nat. Struct. Mol. Biol..

[27] Tucker M., Valencia-Sanchez M.A., Staples R.R., Chen J., Denis C.L., Parker R. (2001). The Transcription Factor Associated Ccr4 and Caf1 Proteins Are Components of the Major Cytoplasmic MRNA Deadenylase in Saccharomyces Cerevisiae. Cell.

[28] Chen J., Chiang Y.-C., Denis C.L. (2002). CCR4, a 3′–5′ Poly(A) RNA and SsDNA Exonuclease, Is the Catalytic Component of the Cytoplasmic Deadenylase. EMBO J..

[29] Traven A., Hammet A., Tenis N., Denis C.L., Heierhorst J. (2005). Ccr4-Not Complex MRNA Deadenylase Activity Contributes to DNA Damage Responses in *Saccharomyces Cerevisiae*. Genetics.

[30] Sanchez-Perez I., Manguan-Garcia C., Menacho-Marquez M., Murguía J.R., Perona R. (2009). HCCR4/CNOT6 Targets DNA-Damage Response Proteins. Cancer Lett..

[31] Zukeran A., Takahashi A., Takaoka S., Mohamed H.M.A., Suzuki T., Ikematsu S., Yamamoto T. (2016). The CCR4-NOT Deadenylase Activity Contributes to Generation of Induced Pluripotent Stem Cells. Mol. Biol. Cell.

[32] Maragozidis P., Karangeli M., Labrou M., Dimoulou G., Papaspyrou K., Salataj E., Pournaras S., Matsouka P., Gourgoulianis K.I., Balatsos N.A.A. (2012). Alterations of Deadenylase Expression in Acute Leukemias: Evidence for Poly(A)-Specific Ribonuclease as a Potential Biomarker. Acta Haematol..

[33] Gutierrez-Camino A., Lopez-Lopez E., Martin-Guerrero I., Piñan M.A., Garcia-Miguel P., Sanchez-Toledo J., Carbone Bañeres A., Uriz J., Navajas A., Garcia-Orad A. (2014). Noncoding RNA–Related Polymorphisms in Pediatric Acute Lymphoblastic Leukemia Susceptibility. Pediatr. Res..

[34] Jiricny J. (2006). The Multifaceted Mismatch-Repair System. Nat. Rev. Mol. Cell Biol..

[35] Aslam A., Mittal S., Koch F., Andrau J.-C., Winkler G.S. (2009). The Ccr4–Not Deadenylase Subunits CNOT7 and CNOT8 Have Overlapping Roles and Modulate Cell Proliferation. Mol. Biol. Cell.

[36] Braun J.E., Huntzinger E., Fauser M., Izaurralde E. (2011). GW182 Proteins Directly Recruit Cytoplasmic Deadenylase Complexes to MiRNA Targets. Mol. Cell.

[37] Drost M., Zonneveld J.É.B.M., van Dijk L., Morreau H., Tops C.M., Vasen H.F.A., Wijnen J.T., de Wind N. (2010). A Cell-Free Assay for the Functional Analysis of Variants of the Mismatch Repair Protein MLH1. Hum. Mutat..

[38] Duan S., Han X., Akbari M., Croteau D.L., Rasmussen L.J., Bohr V.A. (2020). Interaction between RECQL4 and OGG1 Promotes Repair of Oxidative Base Lesion 8-OxoG and Is Regulated by SIRT1 Deacetylase. Nucleic Acids Res..

[39] Mittal S., Aslam A., Doidge R., Medica R., Winkler G.S. (2011). The Ccr4a (CNOT6) and Ccr4b (CNOT6L) Deadenylase Subunits of the Human Ccr4–Not Complex Contribute to the Prevention of Cell Death and Senescence. Mol. Biol. Cell.

[40] Johnson G.E. (2012). Mammalian Cell HPRT Gene Mutation Assay: Test Methods. Methods Mol. Biol..

[41] Wang H., Hays J.B. (2000). Preparation of DNA Substrates for In Vitro Mismatch Repair. Mol. Biotechnol..

[42] Sakamoto M., Iwama K., Sekiguchi F., Mashimo H., Kumada S., Ishigaki K., Okamoto N., Behnam M., Ghadami M., Koshimizu E. (2020). Novel EXOSC9 Variants Cause Pontocerebellar Hypoplasia Type 1D with Spinal Motor Neuronopathy and Cerebellar Atrophy. J. Hum. Genet..

[43] Loveless A. (1969). Possible Relevance of O–6 Alkylation of Deoxyguanosine to the Mutagenicity and Carcinogenicity of Nitrosamines and Nitrosamides. Nature.

[44] Nowosielska A., Marinus M.G. (2008). DNA Mismatch Repair-Induced Double-Strand Breaks. DNA Repair.

[45] O’Brien V., Brown R. (2006). Signalling Cell Cycle Arrest and Cell Death through the MMR System. Carcinogenesis.

[46] Webster M.W., Chen Y.-H., Stowell J.A.W., Alhusaini N., Sweet T., Graveley B.R., Coller J., Passmore L.A. (2018). MRNA Deadenylation Is Coupled to Translation Rates by the Differential Activities of Ccr4-Not Nucleases. Mol. Cell.

[47] Nicholson A.L., Pasquinelli A.E. (2019). Tales of Detailed Poly(A) Tails. Trends Cell Biol..

[48] Lin D.P., Wang Y., Scherer S.J., Clark A.B., Yang K., Avdievich E., Jin B., Werling U., Parris T., Kurihara N. (2004). An Msh2 Point Mutation Uncouples DNA Mismatch Repair and Apoptosis. Cancer Res..

[49] Zhang H., Richards B., Wilson T., Lloyd M., Cranston A., Thorburn A., Fishel R., Meuth M. (1999). Apoptosis Induced by Overexpression of HMSH2 or HMLH1. Cancer Res..

[50] Scherer S.J., Maier S.M., Seifert M., Hanselmann R.G., Zang K.D., Müller-Hermelink H.K., Angel P., Welter C., Schartl M. (2000). P53 and C-Jun Functionally Synergize in the Regulation of the DNA Repair Gene HMSH2 in Response to UV. J. Biol. Chem..

[51] Gazzoli I., Kolodner R.D. (2003). Regulation of the Human MSH6 Gene by the Sp1 Transcription Factor and Alteration of Promoter Activity and Expression by Polymorphisms. Mol. Cell. Biol..

[52] Maragozidis P., Papanastasi E., Scutelnic D., Totomi A., Kokkori I., Zarogiannis S.G., Kerenidi T., Gourgoulianis K.I., Balatsos N.A.A. (2015). Poly(A)-Specific Ribonuclease and Nocturnin in Squamous Cell Lung Cancer: Prognostic Value and Impact on Gene Expression. Mol. Cancer.

[53] Nalla A.K., Williams T.F., Collins C.P., Rae D.T., Trobridge G.D. (2016). Lentiviral Vector-Mediated Insertional Mutagenesis Screen Identifies Genes That Influence Androgen Independent Prostate Cancer Progression and Predict Clinical Outcome. Mol. Carcinog..

