# Supplementary material for: CNOT6: A Novel Regulator of DNA Mismatch Repair
Source: Cells. 2022 Feb 2;11(3):521. doi: 10.3390/cells11030521 (PMC8833972; doi:10.3390/cells11030521)
Supplement: Supplementary file 1 [file cells-11-00521-s001.zip › cells-1553055-supplementary.pdf]

cells

MDPI

SUPPLEMENTARY INFORMATION

Figure S1

A

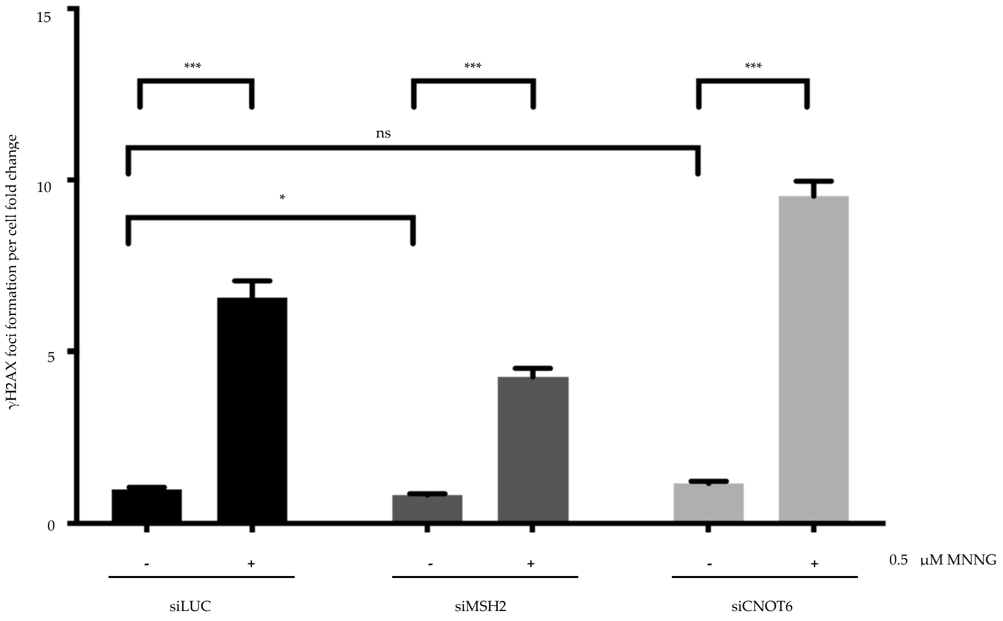

B

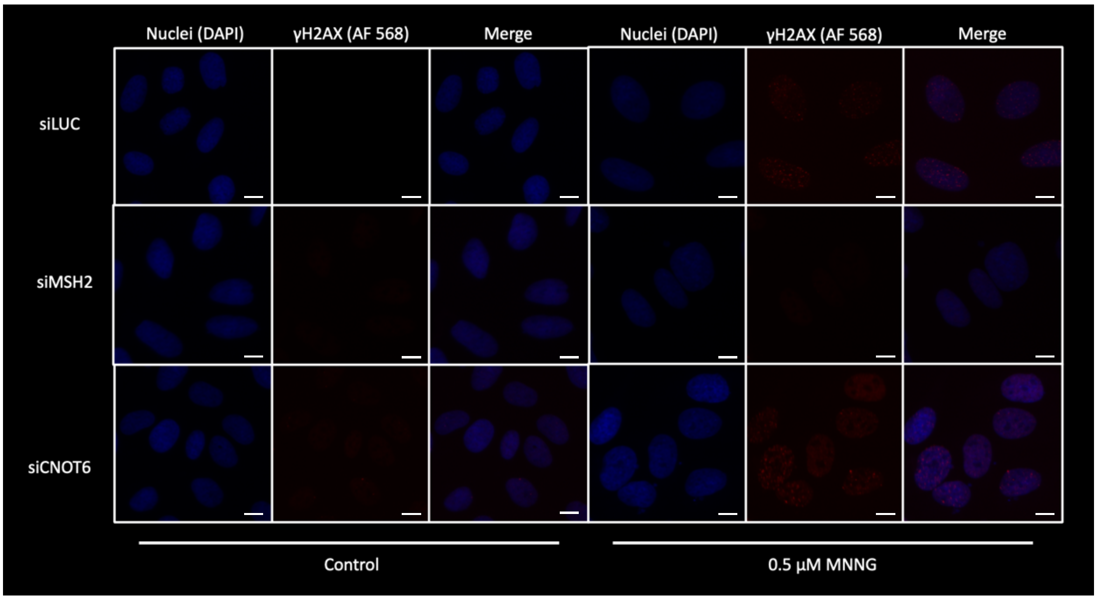

More damage was induced by MNNG treatment in CNOT6-depleted cells through the detection of γH2AX foci formation. (A) Data are shown as mean ± SEM, *n* = 3; statistical significance (**P* < 0.5, ***P* < 0.01, ****P* < 0.001) was determined using unpaired two-tailed Student's *t* test. (B) Representative images of γH2AX foci detection by high-content microscopy.

γH2AX foci detection by high-content microscopy

Human U2OS cells were transfected with siLUC and siCNOT6 followed by the MNNG treatment as described before. 72 h after the treatment, cells were fixed in 4% formaldehyde for 15 min at room temperature, washed three times in PBS for 5 min each and blocked in blocking buffer (1X PBS, 5% normal serum, 0.3% Triton™ X-100) for 60 min. Then cells were incubated with anti- γH2AX antibody overnight at 4°C, followed by Alexa Fluor 568 goat anti-mouse secondary antibody. After wash, cells were incubated with DAPI before subjected to high-content microscopy imaging.

Text S1

Mass spectrometry (MS) analysis

Cell transfection and sample preparation were performed as described above. Samples were then in-gel digested with trypsin and desalted with C18 StageTips. Sequentially, 10% of each sample was subjected to mass spectrometry (MS) analysis using an EASY-nLC 1000 LC system (Thermo Fisher Scientific) coupled with an

Orbitrap Fusion mass spectrometer (Thermo Fisher Scientific). A single analytical column setup using PicoFrit Emitters (New Objectives, 75 μm inner diameter) custom packed with Reprosil-Pure-AQ C18 phase (Dr. Maisch,

1.9-μm particle size, 20 cm column length) was applied. Samples were injected and eluted with a gradient of Solvent B from 2 to 25% at 200 nL/min for 115 min (Solvent A: 100% H2O + 0.1% (v/v) formic acid, Solvent B: 100% acetonitrile + 0.1% (v/v) formic acid). With the nominal resolution setting of 120,000, precursors of full MS scan (m/z 350-2000) were obtained. Then MS/MS spectra of the 10 most abundant multiply charged precursors in the full MS spectrum were acquired at the nominal resolution setting of 60,000 with higherenergy collisional dissociation (HCD). To trigger data-dependent fragmentation events, the minimum signal threshold was 50,000.

The MS data processing was performed in Proteome Discoverer 2.3 (Thermo Fisher Scientific) software using Sequest HT search engine against a concatenated human-specific database (UniProt, March 2019, contacting 20,355 canonical entries). The precursor mass tolerance was set to 10 ppm and fragment ion mass tolerance to 20 milli mass unit (mmu). Carbamidomethylation on cysteine residues was used as a fixed modification and methionine oxidation was used as a variable modification for MS/MS. All spectra were validated by Percolator

Node with the false discovery rate of 1%. For m/z feature recognition from full MS scans, Minora Feature Detector Node in PD 2.3 was used.

Figure S2

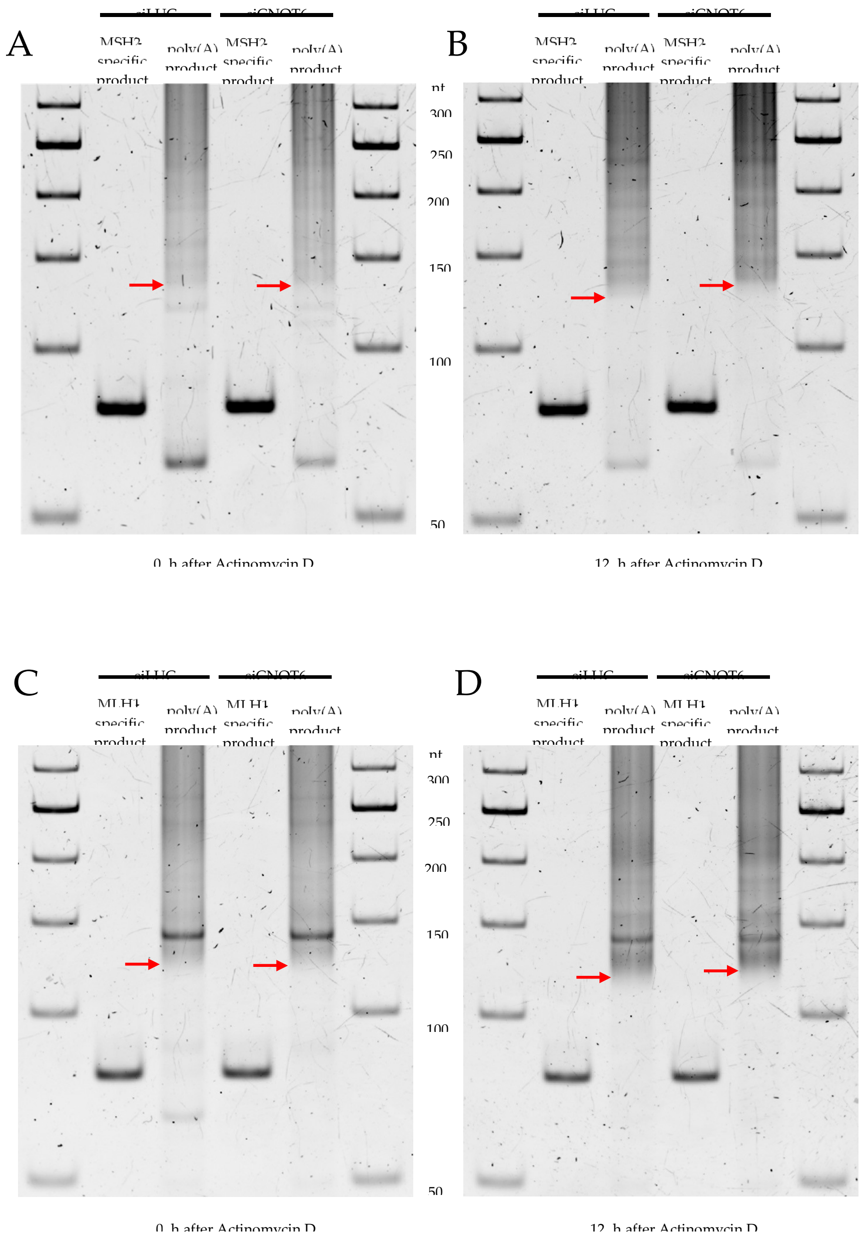

The impact of CNOT6 depletion on poly(A) tail-length. (A) and (B) Representative images of the impact of CNOT6 depletion on poly(A) tail-length of MSH2 at 0 h and 12 h after Actinomycin D treatment, repectively. (C) and (D) Representative images of the impact of CNOT6 depletion on poly(A) tail-length of MLH1 at 0 h and 12 h after Actinomycin D treatment, repectively. Red arrows indicate the bottom of the smear in each lane. *n*=3.

Poly(A) tail-length detection

Human U2OS cells were transfected with siLUC and siCNOT6 as described before. 72 h after transfection, cells were treated with Actinomycin D at a final concentration of 5 µg/ml. Total RNA was isolated 0 and 12 h after treatment. The detection of the poly(A) tail-length was performed according to the manufacturer's protocol (764551KT, Thermo Fisher Scientific). In brief, 1 µg total RNA was used in the G/I tailing step, followed by reverse transcription. After dilution, 5 µl of the reverse transcription sample was used as the template in three-step PCR amplification. PCR

amplification with the gene-specific forward primer and Universal reverse primer, which is complementary to the G/I tail, amplifies the sequence upstream of the poly(A) start site to the end of the poly(A) tails, whereas it amplifies gene-specific sequence with gene-specific forward primer and reverse primer locating immediately upstream of the poly(A) start site. Gene-specific primer sets were designed for MSH2 and MLH1. The MSH2-specific PCR product was 75 nucleotides, while 74 nucleotides for the MLH1-specific PCR product. Half of the PCR reaction (12.5 μl) was loaded per lane on a 6% non-denaturing polyacrylamide TBE gel. Gels were stained with SYBR™ Safe (S33102, Thermo Fisher Scientific) and visualized with ChemiDoc Imaging developer (Bio-Rad). The poly(A) tail-length is the size of poly(A) PCR-amplified products minus the gene-specific products.

Figure S3

A

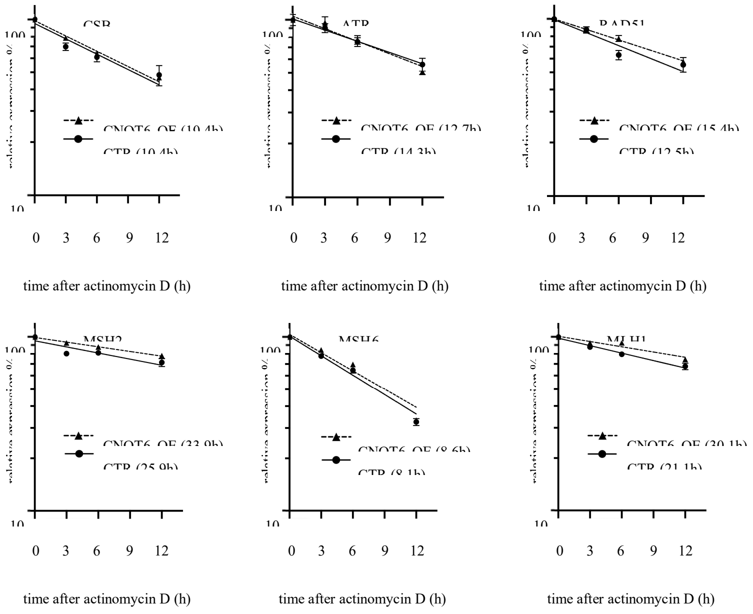

B

Gene.Symbol	half-life time of CTR (h)	half-life time of CNOT6-OE (h)	change of half-life time (h)	ratio (%)
CSB	10.4	10.4	0	0
ATR	14.2	12.7	-1.5	10.56
RAD51	12.5	15.4	+2.9	23.20
MSH2	25.9	33.9	+8.0	30.89
MSH6	8.1	8.6	+0.5	6.17
MLH1	21.1	30.1	+9.0	42.65

Effect of CNOT6 overexpression on various DNA repair mRNA transcripts. (A) U2OS cells transiently overexpressing Flag-tagged-CNOT6. (A) The half-life of *CSB*, *ATR*, *RAD51*, *MSH2*, *MSH6*, and *MLH1*, n ≥ 3. (B) Data from panel A are compared to show the effect of CNOT6 overexpression on the half-life of gene transcripts as mentioned above.
